# Supplementary material for: Neuronal travelling waves explain rotational dynamics in experimental datasets and modelling
Source: Sci Rep. 2024 Feb 12;14:3566. doi: 10.1038/s41598-024-53907-2 (PMC10861525; doi:10.1038/s41598-024-53907-2)
Supplement: Supplementary file 1 — Supplementary Table 1. [file 41598_2024_53907_MOESM1_ESM.pdf]

Supplementary Table 1 – List of all datasets used in the study

**Author(s)**	**Year**	**Dataset Title**	**Dataset URL**	**Database and Identifier**
Churchland et al.	2012	Neural population dynamics during reaching	https://www.dropbox.com/sh/2q3m5fqfscwf95j/AAC3WV90hHdBgz0Np4RAKJpYa?dl=0	10.1038/nature11129
Suresh et al.	2020	Neural population dynamics in motor cortex are different for reach and grasp	https://datadryad.org/stash/dataset/doi:10.5061/dryad.xsj3tx9cm	Dryad Digital Repository, 10.5061/dryad.xsj3tx9cm
Mante et al.	2013	Context-dependent computation by recurrent dynamics in prefrontal cortex	https://www.ini.uzh.ch/en/research/groups/mante/data.html	10.1038/nature12742
Gallego et al.	2022	Local field potentials reflect cortical population dynamics in a region-specific and frequency-dependent manner	https://datadryad.org/stash/dataset/doi:10.5061/dryad.xd2547dkt	Dryad Digital Repository, 10.5061/dryad.xd2547dkt
Kalidindi et al.	2021	Rotational dynamics in motor cortex are consistent with a feedback controller	https://archive.softwareheritage.org/browse/revision/d61decd3cd750147ef098de1041326fd2be07ab2/?path=monkey_analysis/data_neural	10.7554/eLife.67256
Chowdhury et al.	2022	Data from: Area 2 of primary somatosensory cortex encodes kinematics of the whole arm	https://datadryad.org/stash/dataset/doi:10.5061/dryad.nk98sf7q7	Dryad Digital Repository, 10.5061/dryad.nk98sf7q7
